# Consultation on the Libyan health systems: towards patient-centred services

**DOI:** 10.3402/ljm.v8i0.20233

**Published:** 2013-01-24

**Authors:** Reida M. El Oakley, Murad H. Ghrew, Ali A. Aboutwerat, Nabil A. Alageli, Khaldon A. Neami, Rajab M. Kerwat, Abdulbaset A. Elfituri, Hisham M. Ziglam, Aymen M. Saifenasser, Ali M. Bahron, Elhadi H. Aburawi, Samir A. Sagar, Adel E. Tajoury, Hani T.S. Benamer

**Affiliations:** 1 Permanent Mission of Libya to the United Nations Office at Geneva, Switzerland; 2 Department of Respiratory Medicine, Salford Royal, Manchester, United Kingdom; 3 The Libyan National Programme of Organ Transplant at Tripoli, Libya; 4 Department of Orthopeadics, Tripoli Medical Centre, Tripoli, Libya; 5 Department of Family and Community Medicine, University of Toronto, Toronto, Canada; 6 Department of Surgery, South London healthcare NHS Trust, London, United Kingdom; 7 Faculty of Pharmacy, University of Zawia, Libya; 8 Department of Infectious Disease, Central Hospital, Tripoli, Libya; 9 Heart of England Foundation Trust, Birmingham, United Kingdom; 10 Department of Paediatrics, College of Medicine and Health Sciences, United Arab Emirates University, Al Ain, Abu Dhabi, United Arab Emirates; 11 Consultant Pediatrician, Benghazi Medical Centre, Benghazi, Libya; 12 Department of Neurology, New Cross Hospital, Wolverhampton, United Kingdom

**Keywords:** Libya, Health services, Health system, Conference

## Abstract

The extra demand imposed upon the Libyan health services during and after the Libyan revolution in 2011 led the ailing health systems to collapse. To start the planning process to re-engineer the health sector, the Libyan Ministry of Health in collaboration with the World Health Organisation (WHO) and other international experts in the field sponsored the National Health Systems Conference in Tripoli, Libya, between the 26th and the 30th of August 2012. The aim of this conference was to study how health systems function at the international arena and to facilitate a consultative process between 500 Libyan health experts in order to identify the problems within the Libyan health system and propose potential solutions. The scientific programme adopted the WHO health care system framework and used its six system building blocks: i) Health Governance; ii) Health Care Finance; iii) Health Service Delivery; iv) Human Resources for Health; v) Pharmaceuticals and Health Technology; and vi) Health Information System. The experts used a structured approach starting with clarifying the concepts, evaluating the current status of that health system block in Libya, thereby identifying the strengths, weaknesses, and major deficiencies. This article summarises the 500 health expert recommendations that seized the opportunity to map a modern health systems to take the Libyan health sector into the 21st century.

The health care system in Libya has suffered long periods of neglect, poor funding, and lack of development and modernisation programmes. The matter was further complicated by corruption, outdated ideology, and alienation of even the simplest management concepts.

The extra demand imposed during and after the Libyan revolution in 2011 led the ailing health care system to collapse. The Ministry of Health (MoH), in an attempt to establish a modern health care system in Libya, sponsored the National Health Systems Conference (NHSC) that was held in Tripoli, Libya, in August 2012 (1). The NHSC aimed at reviewing the current health systems status, assimilating its problems, and, where there is a consensus, producing recommendation(s) for health system strengthening. We describe the process leading to the debate that took place in the conference and summarise its recommendations.

## Methods

**Fig. 1:**
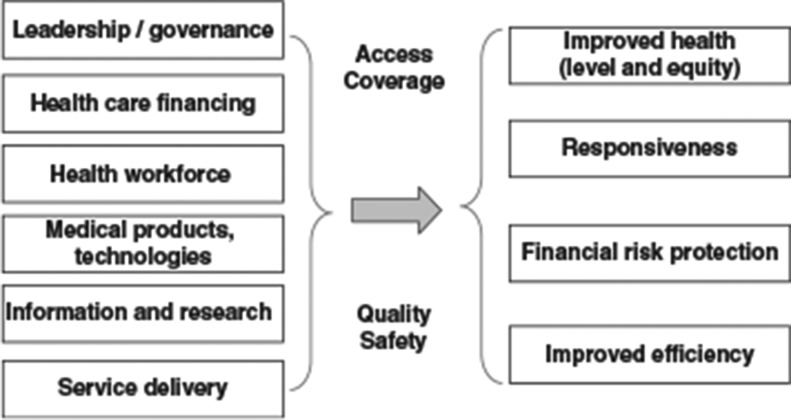
World Health Organisation health system framework. Source: http://www.euro.who.int/en/who-we-are/partners/observatory/health-systems-in-transition-hit-series

The scientific program of the NHSC adopted the World Health Organization (WHO) health care systems framework and used its six building blocks (Health Governance, Health Care Finance, Health Service Delivery, Human Resources for Health, Pharmaceuticals and Health Technology (PHT), and Health Information Service) (2) to structure the debate (Fig. 1). The initial one and half days of didactic lectures and workshops provided the scientific basis of the health systems and addressed the current status of the health service in Libya. These were compared with modern health systems at the international level, including a session on ‘Clinical Governance’ (1). The attendees were a selected group of national health care leaders with a representation from all corners of Libya and all sectors of the health care workforce. The consultation was named LH500 (Libya Health 500), participants were divided into a number of working groups. Participants were encouraged to think about each of the six building blocks, through a structured approach starting with clarifying its objectives, and evaluating the current status of that block in Libya, and thereby identifying its strengths and weaknesses.

Each session was chaired by a national expert and co-chaired by one of the 10 WHO experts. The chairpersons were directed to do the following:

1. Highlight the role of each building block in order to fine-tune the participants with regard to the concept and role of the block under consideration;
2. Present the current status of the index health systems building block in Libya and highlight the anticipated challenges;
3. Guide the discussion towards how to move from the present state of affairs to the desired health system. That was accomplished by presenting up to five questions (or topics for debate) covering the primary areas of concern within the relevant block of the health systems;
4. Identify relevant reading materials and make them available to the participants in advance when possible;
5. Help in drawing the conclusions from the working group’s discussion for presentation at a plenary session.

Towards the end of the half-day session on each block, groups identified potential solutions and priorities for change and, when there was consensus, proposed recommendations. The final report was delivered to the Minister of Health and members of the National Congress Committee on Health on October 1st, 2012.

## Results and recommendations

We summarised the outcome of the discussion and the recommendations under each of the six building blocks of the health systems.

### Leadership and governance

Delegates debated the past and current challenges that have faced the health sector governance and leadership. One of the major problems in Libya, particularly during the previous regime, has been that decision maker(s) have lacked the political-will and have not exercised a genuine effort to improve health care. Corruption was rampant in all government organisations, including health care authorities. The private sector failed to fulfil its potential role in health care delivery or develop any form of partnership with the public sector due to the twisted ideology adopted by the previous Libyan regime.

#### Recommendations

1. To form and empower a supreme council for health (SCH) that has the necessary legislative powers. SCH has to be chaired by the highest possible government authority, preferably the Prime Minister or his/her Deputy as chairperson, and the Minister of Health as Deputy Chairperson. All ministries affected by MoH performance are to be represented by their ministers and/or deputies. The Head of the Parliamentarian Health Supervisory Committee may represent the Parliament. The SCH has the following mandates:
  - To assume the role of the National Crisis Committee with immediate effect.
  - To supervise the National Health Care Strategy addressing the short- and long-term plans of the health care system.
2. Immediate amendment of the outdated and flawed health system laws.
3. Restructure the organisational chart of the MoH, taking the following into account:
  - Separation of the three functions of the health care system under the following independent bodies:
    - National health service and regional health authorities (RHA).
    - Health care finance.
    - Clinical governance body (for setting the standards of services, licensing and inspection of government and private health care facilities and monitoring performance), in addition to the existing Medical Syndicate and the Libyan Board for Medical Specialties. The bylaws of all these governing bodies need to be re-written to remain complementary and in harmony.
  - Decentralisation of medical services to the RHA making them accountable for delivering high-quality health services to local populations. This may be achieved as follows:
    - Appointing a chief executive and a board to manage each RHA. The board should have representatives from patient groups, civil societies and different sub-regions within each RHA. RHA should also have considerable local discretion to determine how local services are organised and delivered. Each RHA may cover a population of 300,000–500,000 individuals.
    - Ensuring that basic primary health and acute services are available within 50 kilometres (unless air ambulance is readily available).
    - Giving the power to each RHA to manage local health care facilities and the mandate to make decisions, and be accountable for the results and outcomes of the clinical services.
    - Delivering the necessary resources to the RHA in a timely manner.
4. Encourage a culture of accountability and transparency in the health sector through the following:
  - Involvement of civil societies and scientific organisations.
  - Enhancing a decision-making process that is evidence-based and subject to regular evaluation by auditing department(s) within MoH and/or other official institutions.
  - Promote ‘Root-Cause-Analysis’ protocols and enhance a ‘no-blame’ culture.
  - The patients and the society should know their rights through the establishment of a patient’s charter and by enhancing health education.
  - Consider the establishment of an independent patient Ombudsman, funded by the government, to be the patients’ own watchdog.
5. Establish Independent Professional Councils to regulate medical and allied healthcare specialties (as outlined above).
6. Establish a National Health Research Council/Centre to regulate and fund health care-related research with the following responsibilities:
  - Selecting research fields and programs that should take priority in funding.
  - Regulation of research to ensure that patient safety, autonomy, and privacy are maintained through a research ethics review and approval system.
7. Regulation of the private sector, taking into account the need for the following:
  - Establishing a licensing system for private health care facilities.
  - Encouraging private health care facilities to comply with the agreed national standards of care.
8. Development of a national program to promote and regulate a public–private partnership (PPP) to ensure the following conditions:
  - PPP remains patient centred.
  - Payments are agreed according to national tariff.
  - Agreed policies that govern PPP with national and international health care providers.
  - Involve the private sector in planning and decision-making for health.

### Health care finance

The government’s responsibility for funding health in industrialised countries varies. At one extreme, government bears little or insignificant responsibility, and it is up to the citizens and/or their employers to take a voluntary insurance or pay directly for their health care (Bismarck model). At the other extreme, health care is provided and paid for through the national tax revenues (Beveridge model). To a great extent, there is no direct correlation between the money spent on health services and the effectiveness of the health care system. For example, the USA spends 17.5% of its gross domestic products (GDP) on health. That is translated into approximately 7,000 US$ per capita per year. European countries, including France, spend 3,000–4,000 US$ per capita per year, while Singapore spends only 800 US$ per capita per year. Yet according to WHO standards of care, the USA ranks number 37 in the world, whereas France and Singapore rank numbers one and six, respectively.

The main problem in health finance in general, and in the Beverage model in particular, is ‘Moral Hazards.’ This stems from the fact that one individual ‘takes’ a decision and another individual ‘pays’ for all related cost. In health care, patients, doctors, and hospitals knowingly or unknowingly commit Moral Hazard in one form or another; for example, patients’ unnecessary visits to hospitals or patients failing to show up for hospital appointment are moral hazards on their behalf. Physicians and hospitals who over-investigate or over-treat patients or send them abroad for treatment, which is available locally, are all moral hazards on behalf of physicians and hospitals.

Medical saving accounts (MSAs), which is the main mechanism of funding health in Singapore, has addressed the dilemma of patient over-utilisation of services (consumer moral hazards) by allowing patients to manage their own MSAs at the point of service delivery, thereby minimising the number of unnecessary clinic visits and hospitalisations. Over-utilisation of resources by health care workers, hospitals, and clinics (moral hazard on the providers’ behalf) remains a difficult problem without an ideal solution. This is particularly true in countries where doctors are paid on the basis of fee-for-service, where defensive medical care is widely practiced, and where the push from industry to use expensive products is relatively uncontrolled. However, reimbursing health care providers using the Diagnosis Related Groups (DRGs) is now widely utilised, and it may be the best option available today. The lack of reliable data on Libya’s funding for health upon which strategic decisions can be made was also highlighted.

#### Recommendations

**Fig. 2:**
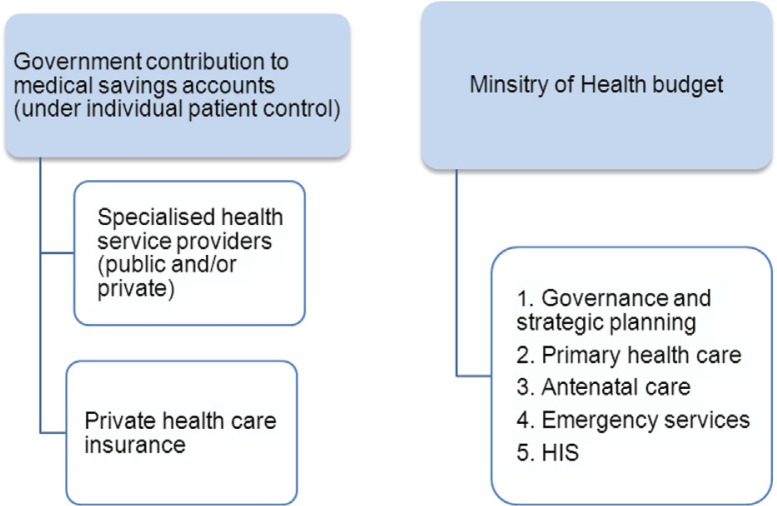
Alternative mechanism for financing health.

1. Universal access to free basic and preventive health care to all Libyans.
2. Major review of the current funding mechanism for health services.
3. Libya’s spending on health should be comparable to upper–middle-income countries.
4. Increase the spending on health care. Some suggested 5.5–6% of GDP as a target by the year 2030. Others recommend that Libyan health funding should be increased by approximately 1% every year over the next 4 or 5 years, aiming at the average health spend in Europe, which is approximately 7.7% of GDP.
5. The mechanism by which the government pays for health has been debated. Suggestions have been made, leading to the following understanding:
  - Primary health care, antenatal care, and emergency services should be provided without any financial implications to individuals.
  - Resources should be distributed across Libya on the basis of need. This should take into consideration the resident population size, the local need for services, and the local costs of providing them. Therefore, the distribution of resources has to be truly equal.
  - Detailed analysis of the current spending on health is required.
  - More information and further consultations may define how to employ funding in order to put the patient in the centre of the health system.
  - The Beverage model is limited with the moral hazards, while the Bismarck model may have religious implications and concerns. There are also concerns that the Bismarck model may lead to unnecessary waste of funds due to introduction of a third party intermediary (e.g. insurance companies).
  - To consider an alternative ‘Libyan-style’ medical insurance (or health savings account) in which the government pays the expected cost of care for the currently ill and the ‘injured’ individuals into a special bank account to be used only for health expenditure by the patient and immediate relatives. This account can be used to pay hospital bills and/or to purchase private health insurance (Fig. 2). This approach might resolve the dilemma of treating the injured once and for all. A similar strategy may be considered for all Libyans in the long term.
  - Fewer participants proposed that the government should continue to pay for health care as a public service in a similar way to funding education and police services today.
  - There is a need to enhance PPP and encourage MoH to buy cost-effective service from the private sector and use finance to encourage high quality services, using case-mix and Diagnosis Related Groups ‘DRGs’.
  - All MoH-affiliated medical institutions should have clinical-negligence insurance that covers all their employees. This can be achieved through a national clinical negligence insurance scheme or through the insurance industry. The latter, however, will prove more costly in the long term.
  - To revive social insurance to its standards of the 1970s and improve it further.

### Human resources for health

Data from the Health Information Centre, MoH, Libya, in 2010 shows a health workforce in Libya of approximately 107,698 (3) (Table 1), but there is a considerable lack of detailed characterisation of training levels and skills mix.

**Table 1:** Number of health care workers (HCW) in Libya in 2010

Discipline	Number
Medical	11,323
Nurses and midwives	40,926
Dentists	3,176
Pharmacists	1,100
Other HCW	16,640
Administrative staff	34,533
Total	107,698

Participants summarised the current problems affecting the effectiveness and responsiveness of the healthcare workforce in Libya as follows:

1. Inconsistent distribution of the health work force, so that some health care facilities are unjustifiably overstaffed while others are severely understaffed.
2. Significant proportions of the workforce, especially within non-medical groups, are generally poorly skilled or unqualified to cope with allocated tasks and responsibilities.
3. There is no credible and/or transparent workforce development programme.
4. Significant proportions of the existing workforce are demoralised and poorly motivated due to poor work environment, low pay, and poor prospects for career progression.
5. Lack of effective professional regulation is a significant factor in the poor performance of professionals at all levels, which compromises the performance of the health care sector and is a major factor in the loss of public confidence in the medical profession.
6. Human resources data are either lacking or inaccurate, making workforce planning difficult.

#### Recommendations

1. Workforce redistribution according to the regional and institutional needs.
2. Detailed analysis of the existing workforce, including numbers, categories, and skills mix is urgently required.
3. Re-categorisation of staff groups and sub-classification depends on qualifications and competencies before redeployment.
4. Providing incentives to staff, either financial or in the form of higher training opportunities, to encourage them to engage in technically demanding or unpopular specialities, such as family practice, biomedical engineering, high-risk areas, and those requiring relocation to geographically remote parts of the country.
5. Workforce strategic planning in order to achieve the following:
  - Establish a robust and comprehensive health workforce database to include numbers, categories and subcategories, skills mix, and qualifications and distribution. Information can be collected through frequent workforce census.
  - Collect information regarding numbers predicted to join the workforce (nursing and medical graduates) and numbers leaving the force (migration and retirement plans).
  - Review current admission policies in medical and paramedical schools to tailor intake for better quality training and reduce predicted waste in medical and paramedical graduates and unjustified large numbers of ‘university leavers.’
  - Find a pathway for those who successfully complete the basic science course and cannot continue to complete the primary medical degree. Offering these students a diploma in basic clinical sciences may save their career and fill vacancies in technical posts such as biomedical engineers, radiographers, and others.
6. Workforce recruitment and retention
  - Introduction of rules for new appointments, such as pre-identified vacancies, job advertisement, job description, formal interviews and introducing the concepts of equal opportunities employers.
  - Improve work conditions, security, empowerment, and involvement of staff in decision making.
  - Motivation of staff through recognition of excellence and promotion based on performance, and creation of opportunities for career advancement.
  - Career planning and shifting roles and responsibilities if and when necessary.
  - Review pension schemes and introduce incentives for continuing work beyond the age of retirement to reduce loss of experienced staff through early retirement.
  - Attract Libyans working abroad to return to work in their homeland.
7. Workforce development
  - There is an urgent need to either update staff skills so that they can competently perform their duties or shift jobs and careers as required, while protecting staff employment rights.
  - There is a need to introduce the concept of continuous professional development and lifelong learning to maintain skills and competencies and adopt advanced courses in professional knowledge and skills.
  - All employees should have annual personal development plans regardless of the nature of their job, with the aim of fulfilling their career ambitions and meeting workplace needs.
  - All employees should be appraised annually.
8. Professional regulation
  - There is a need for the establishment of independent professional regulatory bodies to regulate the medical, dental, pharmaceutical, nursing, and other allied health professions. These bodies should be statutory and non-governmental institutions. They also should work independently yet in tune with each other. Their remits are to regulate the professions through registration, certification, and re-certification. They should also be responsible for issuing professional guidance, ethical standards, and codes of conduct. They should be responsible for assessing the professional fitness to practice when this comes into question. They should monitor medical and paramedical education at both undergraduate and postgraduate levels to ensure that training programmes and university curricula meet the agreed standards and are delivered effectively and reliably.
  - Re-certification should only be possible if the professional has a good, continuous professional development record and demonstrates evidence of safe performance through audit.
9. Although the implementation of the above recommendations could help in restoring public confidence in the health services, media campaigns should be used to build trust between the healthcare professionals and the general public.

### Pharmaceuticals and health technology

The Libyan pharmaceuticals and health technology (PHT) services need urgent modernisation. Current quality assurance systems are inadequate. Drug legislation and regulation is inadequate, and there is no stable or functioning drug regulatory authority with adequate resources and infrastructure. There is a need to implement modern legislation and regulation to circumvent the current supply difficulties and acute shortages.

#### Recommendations

1. Review regulations and legislation related to the importation, production, prescribing and use of PHT and update those regulations to ensure the following:
  - A completed and updated national drug policy should be integrated with the national health strategy.
  - Legislation should form the basis for implementation of the national strategy and policy. It should also represent the first step for regulation of the supply of pharmaceuticals and medical products that may include the development of pharmacy law.
  - Operation procedures related to PHT supply should be rational, professional, transparent, and evidence-based.
  - Systematic, cost-effective selection and a regular update of the national specifications list for pharmaceuticals and other health technology products are recommended.
  - Empowerment of the National Center for Food and Drug Control to become the competent national PHT authority. This authority should be able to monitor and steer the respective policies and legislation. In addition to the required legislative and regulatory actions, the authority needs to be augmented with a professional and efficient infrastructure. It is to be equipped with adequate technical resources and the necessary trained human resources.
  - To develop appropriate products registration and pricing, as well as personnel and facilities licensing procedures to ensure that only legitimate good quality PHT are allowed into the market and sold to the public at competitive prices.
  - To develop an independent Food and Drug Administration (FDA) style pharmaco-vigilance unit and a drug information centre.
  - To develop national treatment guidelines in collaboration with professional bodies and societies.
  - Every health authority and health institution should have a drugs and therapeutics committee that can promote the concepts of essential drugs, its rational use, generics prescribing, and in-service basic training of health professionals in drug management, including a clinical pharmacist for every ward.
2. National policy for drug procurement, storage, and distribution should ensure the following:
  - Essential drugs are readily available and equally distributed in all regions of the country.
  - Procurement may remain centralised to ensure consistency and a more efficient use of resources. However, policy implementation, storage, and service provision should be decentralised.
  - Each local authority or district needs to identify its requirements and priorities and allocate the resources to be used in a cost-effective, efficient, transparent, and equitable manner. Government commitment to the principles of auditing, monitoring, and evaluation is essential. Use of regular indicator-based surveys is required. Satisfaction of users with the pharmaceuticals services is to be measured regularly. A bottom-up model of decision-making is recommended.
  - Drugs and other pharmaceutical products should be stored only in licensed facilities that conform to the international standards of good storage practices. These facilities should be regularly inspected to make sure that those standards are maintained.
  - Drugs inventories at public central, regional, and local drugs storage facilities should be regularly updated. Coordinated central inventory management of distribution and redistribution system will minimise waste.
  - The tendering process for procurement in the public sector should be completely transparent.
  - An accurate health information system (HIS) can provide reliable information about PHT utilisation and distribution and thereby improve decision-making and services delivery.
  - Procurement of medical equipment should be separated from that of the PHT products.
  - PHT-related waste may only be managed by authorised trained personnel in each health facility to reduce risk. The process should be rigorously controlled and transparent.
  - Drugs and medical supply of the private sector should be empowered and monitored. In addition, national commercial agencies need to be enforced, with set responsibilities.
3. The government needs to develop national strategies to reduce corruption and criminal activity and promote inter-disciplinary cooperation between regulatory authorities, police, customs services, and the judiciary to effectively regulate the PHT market and enforce health technology guidelines.
4. Create a supportive environment for the local manufacturing of pharmaceutical products and health technology.
5. Workforce planning and development in the drugs and PHT sector requires the following:
  - Collaboration between the MoH and the Ministry of Higher Education to reform pharmacy schools and improve their outcome.
  - Establishment of postgraduate training programs in fields such as pharmaceutical care, clinical pharmacology, hospital pharmacy, regulatory pharmacy, quality control, pharmaco-vigilance, pharmaco-epidemiology, radio-pharmacy, medical supply management, and pharmaco-economics.
  - Development of partnerships with high standard international universities.
  - Facilitating lifelong continuous professional education.
  - Suppliers’ scientific offices may be encouraged to contribute to health sector development.

### Health information system

Accurate information is the basis for all strategic decision-making across the health care system, at both local and national levels. There is no clear national HIS strategic plan, and there is a severe shortage of trained information and communication technology (ICT) staff that can develop local and national ICT systems. The legislation necessary for underpinning any health care information management is lacking, particularly legislation that governs data protection and disclosure. There are no national policies or guidelines on data collection and management.

#### Recommendations

1. Establishment of a national information technology steering committee to oversee the development of HIS for the entire health care system. The committee remit is as follows:
  - Developing a national ICT vision, strategy, and plan.
  - Overseeing the implementation of the agreed plans.
  - Coordinating with the regulatory body to facilitate the collection of information related to monitoring performance and quality assurance.
  - Proposing national policies and guidelines for data collection and management.
2. Identification of a clear funding stream for the development of IT resources and infrastructure.
3. Recruitment and training of ICT staff to work side by side with clinicians to develop and manage local and national health IT systems, train clinical staff, and develop their skills for the effective utilisation of HIS. Trained staff are also needed for compilation, analysis, and reporting of health information.
4. Collect high quality national data through the following:
  - Use of nationally agreed data collection standards and coding systems, operation codes, unique patient numbers, and staff code identifiers.
  - Establish a system to audit and monitor data quality to ensure that it conforms to nationally agreed standards.
  - Propose legislation to govern data protection and disclosure. This should be enforced and monitored by an independent regulatory body to give patients the confidence that their data is safe and will only be used for their benefit.
  - Comparative data about performance of health organisation should be collected and made available in the public domain to be used as a driving force for quality improvement and help in regaining public confidence in the health care system.
  - The following points should be considered in order to build a successful local HIS:
    - Open systems built up over time: starting as small projects has often been more successful than large-scale costly IT projects.
    - Preference should be given to agreeing and controlling systems locally, rather than imposing systems from above.
    - The goal should be to continuously enhance systems to improve clinical decision-making and performance outcome.
5. Development of ICT systems through the following:
  - Setting targets and time scale.
  - Involvement of staff (users) through questionnaires, user groups, and local workshops.
  - Each health facility needs to establish an ICT department.
6. There is a need for the development of national registers for some diseases or disease groups, such as cardiovascular diseases, cancer, and trauma.
7. Consideration should be given to integrating or linking health care information systems with other national ICT systems, such as social services. However, such data exchange or integration should be governed through implementing ICT communications standards for data exchange between organisations.

### Health service delivery

A good health care provision delivers patient-centred, effective, and high-quality health interventions to those who need them, when and where they are needed, without waste of resources. The current health care service in Libya does not conform to any quality standards and is not regulated or monitored. This leaves patients lost, unable to access the service, and not confident in the care that they receive.

#### Recommendations

1. Re-orientation of health service delivery to the patient, to guarantee good access, reliability of care and universal coverage.
2. Reconfiguration of the national health care system into three main functions—health care finance, service delivery and monitoring and quality assurance—so that each function works independently but also in harmony with the others.
  - Health care finance manages health care funds and contracting health care providers, including public health care institutions and private facilities through PPPs.
  - The monitoring and quality assurance body has the responsibility of setting the standards that health care providers have to meet. This body implement the regulation and monitoring system and produce annual reports about the service quality of performance.
  - Service delivery within the public health care system should be re-designed to provide decentralised, comprehensive coverage.
  - Private health care facilities should be encouraged to become a serious partner in supplementing the public health care system.
3. To develop primary care services, including upgrading infrastructure and building new health care centres to ensure universal coverage for essential services and:
  - Regulate access to the health care network so that non-urgent services are delivered locally within primary health care centres or referred onwards from these centres to secondary care when appropriate.
  - Consider a national electronic registration system.
  - Arranging primary health care centres so that they work together in conglomerates and in collaboration with local polyclinics.
  - Introducing an incentive system to attract Libyan doctors to specialise and work in primary care and other much needed sub-specialties.
  - De-centralisation of health care services so that RHAs become responsible for ensuring comprehensive health care to the local population including preventive, curative, palliative, rehabilitation, and health promotion.
  - Re-organising secondary and tertiary care to cover the appropriate catchment areas.
  - Patient choice could be safeguarded through a flexible referral system, overlap of catchment areas, and regulated partnership with the private sector.
4. Good HIS within the healthcare network to ensure continuity of care and readily accessible patient records to support clinical decision-making.
5. Development of national programmes to deal with major public health issues, including cardiovascular disease prevention, cancer screening, smoking cessation, road safety, and health-related environmental issues.
6. Purchasing heath service from international academic institutions with proven track records in health care delivery can only be considered if all Libyans are covered by a comprehensive health insurance scheme.

## Conclusions

The 500 Libyan health experts believe that despite the time constraints, the most urgent and essential difficulties facing the Libyan health systems have been identified. Potential solutions and direct recommendations have also been suggested, with alternative options on occasion. These experts recommend the establishment of a National Health System Task Force to carry forward these recommendations.

It is essential that the General National Congress and the future Libyan parliament as well as current and future governments grant the necessary legislative powers to the National Health System Task Force and the MoH to introduce the necessary administrative changes and help implement the recommendations on a short, medium and long-term basis.
